# Shedding light upon various tools to assess orthorexia nervosa: a critical literature review with a systematic search

**DOI:** 10.1007/s40519-019-00735-3

**Published:** 2019-06-21

**Authors:** Martina Valente, Elena V. Syurina, Lorenzo Maria Donini

**Affiliations:** 1 Faculty of Science, Athena Institute, Vrije Universiteit Amsterdam, Amsterdam, The Netherlands; 2 Experimental Medicine Department, Sapienza University of Rome, Rome, Italy

**Keywords:** Orthorexia nervosa, Diagnosis, Psychometrics, Eating behavior, Assessment

## Abstract

**Aim:**

The aim of this literature review was to identify the tools developed and used to assess orthorexia nervosa (ON).

**Methods:**

A systematic search was executed in PubMed, Biomed Central, and PsycINFO. The final list included 70 articles that were critically analyzed.

**Results:**

A total of six tools were reported to assess ON: the BOT, the ORTO-15, the EHQ, the DOS, the BOS, and the TOS. The tools were based upon different conceptualizations of ON and of its diagnostic criteria. Although they were different, all the conceptualizations derived from the initial definition of ON provided by Bratman in 1997. None of the methodologies adopted for tool construction considered end users or client perspectives and, when carried out, the validations of the tools were fragmented and often based on specific populations.

**Conclusion:**

This study may be a starting point for the construction of a new diagnostic tool for ON. Starting from the methodological weaknesses identified by this review, it was possible to derive some suggestions for future research: (a) developing a modern re-conceptualization of ON, comprehensive of end-user perspectives; (b) adopting qualitative data collection techniques to gain insights into how to diagnose ON; and (c) actively involving diverse stakeholders for constructing a new tool.

**Level of evidence:**

Level of Evidence: I, systematic review.

## Introduction

The term orthorexia nervosa (ON) was first proposed by Bratman in 1997 to define a pathological fixation to eat proper food [1]. Since then, not only has ON had a great media success, but it has also been the subject of many scientific studies. Orthorexia nervosa is characterized by an exasperated selection of food based on beliefs of health aspects [2]. ON starts as an innocent desire to overcome illness or to improve health, but over time, it becomes a strict self-imposition of a dietary regimen, which also feeds a sense of superiority over those who eat unhealthy food [1]. This behavior can be considered ON when the individual spends most of the time planning, purchasing, and eating healthy meals [1], and when obsessive thoughts, compulsive behaviors, self-punishments, and escalating restrictions appear [3]. This disordered eating pattern has negative consequences for the physical health and social life of the individual. The physical health may worsen, because entire categories of food may be avoided, causing nutritional deficiencies [2, 4, 5]. The social life may worsen, as this avoidance of food categories may influence social behavior, causing social isolation and disruption of social relationships [5, 6].

The literature review recently conducted by Cena et al. [7] reports that no official set of diagnostic criteria for ON exists. Consequently, some studies adapted to orthorexia the DSM criteria for anorexia nervosa (AN), avoidant/restrictive food intake disorder, and body dysmorphic disorder, while other studies developed specific diagnostic criteria for orthorexia [7]. This adoption of different diagnostic criteria has led to the development of different diagnostic tools, which report prevalence rates ranging from 1 to 90% [8, 9]. Despite the most used tools have been the ORTO-15 and the orthorexia self-test (BOT), the Dusseldorf Orthorexia Scale (DOS) and the Eating Habits Questionnaire (EHQ) have also been used in some studies [7]. Furthermore, psychometric concerns have been raised about these tools and new tools have been developed recently with the intent to overcome these criticisms [5]. The result is a large number of fragmented, often just loosely connected tools to diagnose ON, each based on its own conceptualization of ON and its own interpretation of diagnostic criteria.

The lack of an agreement on what orthorexia nervosa is and on how it can be diagnosed contributes to its non-recognition as a psychiatric disorder by the international psychiatric classifications such as DSM and ICD. However, before we reach such an agreement, a critical analysis is needed of the existing diagnostic tools for ON and of the diagnostic criteria on which these tools are based. Although there has been a recent review about ON [7], it did not strictly focus on diagnostic tools and on their critical comparison. Furthermore, a new tool has recently been developed, i.e., the Teruel Orthorexia Scale [10], which was not included in the review by Cena et al. Having an overview of the basis of the tools, insights in the way they were developed and applied may shed light on their strengths and limitations and provide the base for development of a unified approach to ON.

### Research aim

The aim of this literature review was to map and present an in-depth critical analysis of the existing tools that were created and used to assess ON. This was achieved by reviewing the various conceptualizations of ON used in the tools, mapping the methods utilized for development and validations of different tools, and identifying the strengths and limitations of these tools.

## Methods

### Search procedure

**Fig. 1:**
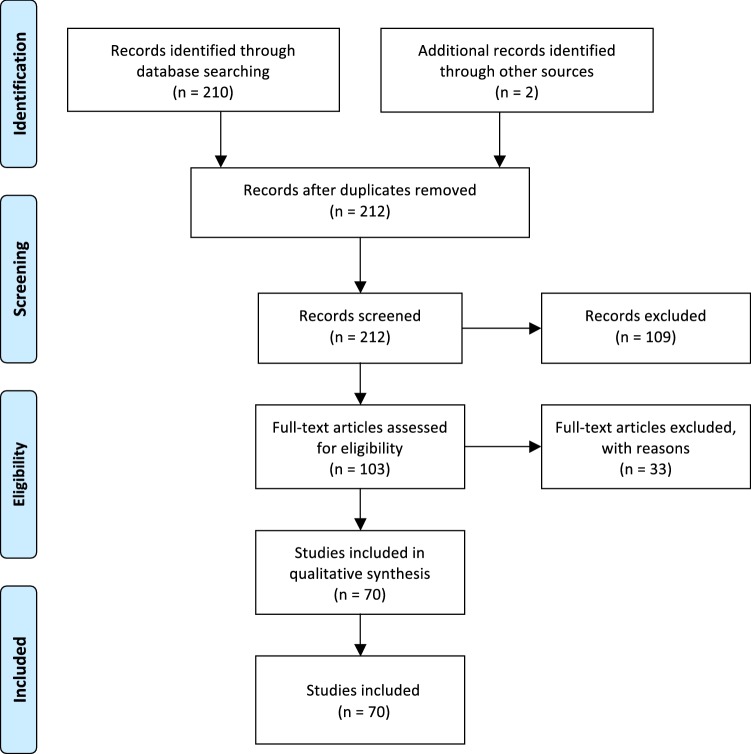
Flow diagram of articles identification and inclusion

The search for relevant articles was conducted via two routes, due to the broad scope of this study. First, PubMed, Biomed Central, and PsycINFO databases were systematically searched. No restrictions were made regarding the publication date, because ON is a relatively new disorder. The search syntax used was: “orthorexia OR orthorexic NOT review”. This was done to exclude literature reviews and to include only original articles. The second route included a manual search of grey literature and/or books that possibly presented the development of a diagnostic tool for ON (e.g., the book *Health Food Junkie*). This was done using the search engine Google and Google Scholar using the keywords: orthorexia and orthorexic. The aim was to identify the books or non-peer reviewed articles that possibly developed tools or instruments to assess ON. This was done, because ON is very popular outside the scientific community and information about ON can be found on the internet or in books. The literature search was conducted according to the seven steps reported on the Cochrane Handbook of Systematic Reviews of Interventions [11] and following procedures described in PRISMA statement. A flowchart describing these processes is reported in Fig. 1.

### Inclusion and exclusion criteria

In line with the aim of the review, inclusion criteria were: (a) study that develops a tool to assess ON, that validates a tool to assess ON, that adapts/translates a tool to assess ON, or that uses a tool to assess ON; (b) full-text available; (c) English, Italian, or German language. The choice of languages was determined by the languages spoken by the authors.

### Screening and study selection

The initial search produced a total of 212 articles. Seventy-three duplicates were removed, together with 36 articles, whose full text was not available, or that did not meet the language requirements. The abstracts of the remaining 103 articles were screened, and 33 articles were removed, because they did not meet the inclusion criteria for this review. A total of 70 articles were obtained in the end, which were divided in two groups, namely, studies that developed, validated, or adapted diagnostic tools for ON (*n* = 22), and studies that used diagnostic tools to diagnose ON (*N* = 48).

### Data analysis

The analysis was initially conducted by separating studies that developed, validated, and adapted a tool to assess ON, from studies that used a tool to assess ON. The first category of articles was critically read with the intention of extrapolating all the important information. The second category was read with the intention of exploring how the tools were used. Information extrapolated by the first category of articles was: (a) title, (b) publication year, (c) author, (d) aim, (e) context, (f) conceptualization of ON, (g) characteristics of ON, (h) diagnostic criteria (i) methods, (j) psychometric properties, and (k) results. Information extrapolated by the second category of articles was: (a) title, (b) publication year, (c) author, (d) aim, (e) context, (f) conceptualization of ON, (g) characteristics of ON, (h) tool used, (i) methods (j) critiques to the tool, and (k) results. A critical comparison of the information obtained was finally conducted and all the information regarding a specific tool were integrated together.

## Results

A detailed overview of the most important information extrapolated by the articles is reported in Table 1. Of the 70 articles analyzed, 22 articles refer to studies that developed, validated, or adapted diagnostic tools for ON. Of these 22 articles, seven articles refer to studies that developed original tools, this meaning that the remaining 15 articles refer to studies that validated or adapted existing tools. Forty-eight articles refer to studies that used diagnostic tools to diagnose ON.

**Table 1:** Table reporting the most important information for each diagnostic tool

	Author	Year	Country	ON conceptualization	No. of adaptations	No. of studies that used the tool	Main criticisms
BOT	Bratman and Knight	2000	USA	Obsession over healthy eating, which seems to be acquiring the characteristics of an ED	2	7	Lack of validation [12–14]
ORTO-15	Donini et al.	2004, 2005	Italy	Eating behavior disorder, characterized by a combination of eating, behavioral, and obsessive-phobic personality traits	10	32	Overestimation of ON prevalence [9, 15–18] Weak psychometric properties [9, 15, 19, 20]
EHQ	Gleaves, Ambwani and Graham	2013	USA	Overwhelming preoccupation on eating healthfully	–	5	Lack of criterion-related validity [21]
DOS	Barthels, Meyer, and Pietrowsky	2015	Germany	A possibly pathological fixation on a healthy diet	1	5	Inability to differentiate between anorexic and orthorexic patients [22]
BOS	Bauer et al.	2018	Spain	Pathological fixation on healthy food intake	–	–	–
TOS	Barrada and Roncero	2018	Spain	An extreme or excessive preoccupation with eating food believed to be healthy	–	–	–

The first category of articles, namely, those that developed, validated, or adapted diagnostic tools for ON, were published between 2000 and 2018. The countries where these studies came from were: Spain (*n* = 4), USA (*n* = 3), Germany (*n* = 3), Austria (*n* = 2), Italy (*n* = 2), Turkey (*n* = 2), Poland (*n* = 2), Australia (*n* = 2), Brazil (*n* = 1), and Hungary (*n* = 1). The second category of articles, namely, those that used a tool to diagnose ON, was published between 2006 and 2018. The countries, where these studies came from were: Italy (*n* = 8), Turkey (*n* = 7), USA (*n* = 7), Poland (*n* = 6), Germany (*n* = 6), Sweden (*n* = 2), UK (*n* = 2), Spain (*n* = 2), Portugal (*n* = 2), Hungary (*n* = 2), India (*n* = 1), Austria (*n* = 1), and Greece (*n* = 1). The majority of the studies that developed and used tools to diagnose ON used quantitative methodologies for data collection.

Below is reported a description of the tools created to diagnose ON. The tools will be presented following an historical timeframe. The information will be presented in the following order: (a) year and country of origin; (b) conceptualization of ON; (c) diagnostic criteria considered; (d) methodology employed; (e) characteristics of the tool; (f) adaptations; (g) adoption of the tool from other studies; and (h) criticisms of the tool.

### Orthorexia self-test

#### Year and country of origin

The orthorexia self-test developed by Bratman (BOT) was the first questionnaire created to assess ON. It was developed at the beginning of the 2000s by US physician Steven Bratman and was first presented in his book *Health Food Junkies* [23].

#### Conceptualization of orthorexia nervosa

The conceptualization of ON on which the tool is based defines ON as an “obsession over healthy eating, which seems to be acquiring the characteristics of an ED” [23].

#### Diagnostic criteria considered

Although Bratman mentioned no diagnostic criteria, the BOT was based on some characteristics of the disorder that the author identified in his daily practice as an alternative medicine practitioner. These characteristics are: (a) spending more than 3 hours per day thinking, cooking, shopping, and reading about food; (b) planning future meals; (c) caring more about the healthiness of food than the pleasure of eating; (e) diminished quality of life; (f) getting stricter with the diet; (g) decreasing social experiences with food; (h) feeling of superiority/increasing self-esteem; (i) feeling of guilt; (j) social isolation; (k) sense of control [23].

#### Methodology employed

No methodological construct used to develop the tool was reported. For this reason, Bratman defined the BOT an “informal” test [23] or, more recently, even a “non-existent” test [3].

#### Characteristics of the tool

The tool consists of 10 yes/no questions, the answer of which allows identifying ON. Precisely, if an individual answers “yes” to two/three questions, he/she has at least a touch of ON; if an individual answers “yes” to four questions, he/she is in trouble; and if an individual answers “yes” to all the questions, he/she needs help [23]. The original version of the BOT lacks of validation; therefore, no psychometric properties were found in the literature.

#### Adaptations

The BOT was adapted to other languages, i.e., German and Swedish [13, 24]. The German version of the BOT, the ORTHO-10, underwent a validation process in Germany [25]. The validation excluded one item from the questionnaire and suggested a two-factor model, the factors being “eating disorders specific” and “orthorexia specific” [25]. The internal consistency was shown to be better for the first factor and than for the second one (*α* = 0.71 and *α* = 0.57, respectively), with a total Cronbach alpha of *α* = 0.73 for the random sample [25].

#### Adoption of the tool by other studies

Despite the lack of validation, the original BOT was used by four studies [12, 14, 26, 27]; the Swedish version of the BOT was used by one study [13]; and the ORTHO-10 by two studies [24, 28]. The studies were aimed at exploring risk factors for ON and exploring associations between ON and other phenomena. None of the studies was aimed at identifying the prevalence of ON in a certain group or area; therefore, more than percentages obtained by the tool, the studies considered differences between groups.

#### Criticisms of the tool

The main criticism raised towards this tool was the poor clinical utility due to the lack of validation [12, 14, 26].

### ORTO-15 test

#### Year and country of origin

The ORTO-15 test has been the most used tool to assess ON. It was created in the years 2004–2005 by an Italian research group [2, 29].

#### Conceptualization of orthorexia nervosa

The conceptualization of ON on which the tool was based defines ON as an “eating behavior disorder, characterized by a combination of eating, behavioral, and obsessive-phobic personality traits” [2].

#### Diagnostic criteria considered

The diagnostic criteria considered by the authors to diagnose ON were the presence of health-fanatic eating habits, and the presence of obsessive–compulsive habits or phobia [2].

#### Methodology employed

The test was derived from the BOT, but the authors modified the verbal aspects of some items and added questions reflecting the obsessive–compulsive traits.

#### Characteristics of the tool

The tool consists of 15 multiple-choice questions, which inquire about the cognitive-rational area, the clinical area, and the emotional area. The answers are based upon a 4-point Likert scale (always, often, sometimes, never). Questions that reflect an orthorexic tendency are scored one point, whereas four points are assigned to those showing normal eating habits. Therefore, higher scores indicate more moderate orthorexic tendency, while lower scores indicate ON. The threshold initially established by the authors was 40; however, studies that considered a threshold of 35 can also be found. A validation followed the construction of the tool, which confirmed a three-factor model, and reported a sensitivity of 100%, a specificity of 73.6%, a positive predictive value of 17.6%, and a negative predictive value of 100% [29].

#### Adaptations

Several adaptations to other languages followed the construction of the ORTO-15. Two adaptations were done to Turkish [30, 31]. The first adaptation did not evaluate the psychometric properties of the translated tool, while the second one executed a validation process, which reduced the number of items to 11, confirmed a three-factor model, obtained a Cronbach alpha of *α* = 0.62, and defined a threshold value of 27 [31]. The ORTO-15 was then adapted to Portuguese [32]. In this case, the authors confirmed a three-factor model and removed three items, thus obtaining a 12-item questionnaire with a Cronbach alpha of *α* = 39. Following, the ORTO-15 was adapted to Hungarian [33]. This adaptation confirmed a single-factor model and implied again the removal of three items, with the obtainment of a Cronbach alpha of *α* = 0.82. Two polish adaptations followed [34, 35], with the first one maintaining nine items and confirming a Cronbach alpha of *α* = 0.64, and the second one maintaining all 15 items and confirming a Cronbach alpha of *α* = 0.78. The ORTO-15 was then adapted to German [36]. This adaptation removed six items and confirmed a 9-item questionnaire with a Cronbach alpha of *α* = 0.67 and a single-factor structure. The adaptation of the ORTO-15 to English was carried out by two Australian research groups [37, 38]. The first group maintained nine items and calculated a Cronbach alpha of *α* = 0.73, while the second one maintained just seven items, calculated a Cronbach alpha of *α* = 0.83, and confirmed a single-factor model. Finally, an adaptation to Spanish was carried out [39, 40], which confirmed a three-factor structure for an 11-item questionnaire, confirmed a Cronbach alpha of *α* = 0.75, and established a threshold of 25.

#### Adoption of the tool by other studies

The ORTO-15 and its adaptations were used by 32 studies. The aims of these studies can be grouped in five main categories: (a) identification of risk factors for ON; (b) identification of cross-cultural differences; (c) investigation of the relationship between ON and AN or obsessive–compulsive disorder (OCD); and (d) prevalence of ON. Two case studies adopted the ORTO-15 to assess the presence of orthorexic traits [20]. Many of the studies were conducted on a sample of students (*n* = 15) [15, 18, 40–52], while other studies focused on risk groups, such as eating disorders patients (*n* = 3) [16, 44, 51], athletes or gym attendees (*n* = 3) [19, 53, 54], dietitians (*n* = 2) [55, 56], vegans/vegetarians (*n* = 1) [57], yoga practitioners (*n* = 1) [58], and artists (*n* = 1) [59].

#### Criticisms of the tool

Several criticisms were raised towards the ORTO-15 and its adaptations. First, it was accused to overestimate the prevalence of ON [16, 18], because it incorrectly identifies dieting as harmful, without also confirming accompanying pathology [9, 46]. Second, validity and reliability of the tool were also questioned [15, 55], together with its internal consistency [60]. Finally, the ORTO-15 was accused to not be based on the most recent diagnostic criteria developed by Dunn and Bratman [71]. Despite that, however, some authors acknowledged the ORTO-15 being at present the only well accepted method of screening for symptoms of ON [15, 55].

### Eating Habits Questionnaire

#### Year and country of origin

The Eating Habits Questionnaire (EHQ) was developed in 2013 by Gleaves, Ambwani, and Graham in the USA [8].

#### Conceptualization of orthorexia nervosa

The conceptualization of ON on which the EHQ is based defines ON as an “overwhelming preoccupation on eating healthfully” [8].

#### Diagnostic criteria considered

The diagnostic criteria considered for the tool construction were extrapolated from the analysis of Bratman and Knight’s case studies.

#### Methodology employed

The construction of the tool started from an initial 160-item pool. Additional ten items required participants to rank the importance of five qualities. Following, four advanced graduate students in clinical psychology dealing with ON in their practice assessed the degree to which the content surveyed by the EHQ captured the construct of ON. Only items that all four raters agreed upon were maintained. This process initially resulted in 59 items. After the validation process, the final tool was reduced to 21 final items.

#### Characteristics of the tool

The answers to the 21-item questionnaire need to be ranked on a Likert scale (from “False, not at all true” to “Very true”). The areas inquired by the questionnaire are knowledge of healthy eating, problems associated with healthy eating, and feeling positively about healthy eating. The validation that followed confirmed a three-factor model, with an internal consistency of *α* = 0.90, *α* = 0.82, and *α* = 0.86 for the three factors, respectively.

#### Adoption of the tool by other studies

Five studies that used the EHQ were found by the current review [20, 60–63]. The main aims of these studies were (a) to assess ON correlates with personality traits, (b) to assess the relation between ON and exercise, and (c) to investigate the influence on ON of ethical or non-ethical motives for following special diets. The majority of the studies was conducted on a sample made up of students (*n* = 3).

#### Criticisms of the tool

Except for the lack of criterion-related validity [21], no criticisms were raised towards this specific tool.

### Dusseldorf Orthorexia Scale

#### Year and country of origin

The Dusseldorf Orthorexia Scale (DOS) is a tool developed in 2015 by Barthels, Meyer, and Pietrowsky in Germany [64].

#### Conceptualization of orthorexia nervosa

The authors conceptualized ON as “a possibly pathological fixation on a healthy diet”.

#### Diagnostic criteria considered

The diagnostic criteria considered for the tool construction were extrapolated from the analysis of Bratman and Knight’s case studies.

#### Methodology employed

The quantitative methodology employed was based upon a multi-level item and factor-analytical selection process, with an evaluation on a sample of 1340 subjects.

#### Characteristics of the tool

The tool is made up of ten questions aimed at measuring orthorexic eating behaviors. A 4-point Likert scale is applied, which goes from “This applies to me” (four points) to “This does not apply to me” (one point). Higher scores indicate the presence of ON. The threshold value that has to be considered is 30, while scores ranging between 25 and 29 indicate risk for ON. The validation of the tool confirmed a single-factor model and an internal consistency of *α* = 0.84.

#### Adaptations

After its construction and validation in German, it was validated in English (E-DOS) [65]. The E-DOS confirmed a Cronbach alpha of *α* = 0.88 and showed that the elimination of any of the ten items would not increase this value. Furthermore, it confirmed a single-factor model.

#### Adoption of the tool by other studies

Five studies conducted in Germany used the DOS to evaluate the presence of ON from 2016 to 2018 [22, 66–69]. The aims of these studies revolve around investigating the association between ON and special diets or AN, and identifying the prevalence of ON.

#### Criticisms of the tool

Only one criticism has been made of the DOS, that is to say that in patients suffering from AN, it does not seem to be able to differentiate between anorexic and orthorexic patients [22].

### Barcelona Orthorexia Scale

#### Year and country of origin

The Barcelona Orthorexia Scale (BOS) was developed in 2018 by Bauer et al., in Spain.

#### Conceptualization of orthorexia nervosa

The conceptualization of ON on which the tool construction was based defines ON as a “pathological fixation on healthy food intake” [70].

#### Diagnostic criteria considered

With regard to the diagnostic criteria, the tool was based upon the latest diagnostic criteria developed by Dunn and Bratman [71] and the available scientific literature on ON.

#### Methodology employed

The methodology adopted to develop the tool was the Delphi method, which consisted in an iterative process in which Spanish and English experts in the field of eating disorders gave their opinion about ON, more than once. The process was anonymous and experts received feedback after each round through group statistical response. The experts assessed for each item: (a) representativeness of the specific content area of ON; (b) clarity; and (c) possible observations. Only experts who participated in the previous round were invited to the next one. In the second and third rounds, experts had the possibility to see group statistical response, expressed through median and interquartile range. Consensus was reached when more than 50% of responses agreed with a statement. The final result was a questionnaire that included 64 items investigating six content areas: (a) cognitive; (b) emotional; (c) behavioral; (d) negative consequences on health; (e) negative consequences on social and academic functioning; and (f) differential diagnosis. The BOS lacks validation; therefore, no psychometric properties are available to be consulted.

#### Criticisms of the tool

No studies were found that used the BOS to assess the presence of ON; therefore, no criticism was found to this tool. However, the authors themselves identified some limitations. First, Spanish experts were significantly less knowledgeable about ON than the English ones. Second, not all experts were specifically dealing with ON, but some of them were dealing with eating disorder in general. Third, many of the participants were working in the field; therefore, 57% of them did not publish papers on ON. Finally, participants were contacted by looking at authors of scientific articles; thus, it may be that some of them were co-authors, being students or statistical consultant [70].

### Teruel Orthorexia Scale

#### Year and country of origin

The Teruel Orthorexia Scale (TOS) is the latest tool developed to assess ON. It was created in 2018 by Barrada and Roncero, in Spain.

#### Conceptualization of orthorexia nervosa

The conceptualization of ON on which the authors based on the TOS comes from the latest publication of Steven Bratman [3] and defines ON as “the pathological aspect of Orthorexia”, being “an extreme or excessive preoccupation with eating food believed to be healthy” [10]. This dualistic conceptualization of ON implies also the presence of a “healthy Orthorexia”, which instead is an approach to healthy nutrition that is not pathological.

#### Diagnostic criteria considered

The diagnostic criteria considered by the authors were based on an extensive literature review, after which two authors independently developed a battery of items that identify ON.

#### Methodology employed

The first step for the creation of the tool was the development of a pool of 93 items characterizing ON. After deleting duplicates, 46 items remained, which were then grouped independently by the authors. With the aim to create a questionnaire with mutually exclusive content, redundant items were deleted, until a final version of 17 questions. A validation process followed, which confirmed a two-factor model (i.e., healthy orthorexia and orthorexia nervosa) and an internal consistency of *α* = 0.85 for the healthy orthorexia and of *α* = 0.81 for orthorexia nervosa.

#### Criticisms of the tool

No studies were identified by the current review that used the TOS to assess the presence of ON. Therefore, no criticisms were identified for this tool.

## Discussion

The objective of this review was to identify which tools have been developed and used to assess orthorexia nervosa. The results report that six diagnostic questionnaires have been constructed from 2000 until now, namely, the BOT, the ORTO-15, the EHQ, the DOS, the BOS, and the TOS. Some of them have been widely used to assess ON worldwide, while others have never been used. Some tools received criticisms from the authors who used them, these criticisms being psychometric flaws, overestimation of ON prevalence, and lack of agreement upon a shared version of diagnostic criteria.

By comparing the areas investigated by the tools, it was possible to draw up a list of ten main themes: (a) spending a lot of time thinking, purchasing, preparing, and planning healthy meals; (b) paying attention to calories and healthiness of food more than the pleasure of eating; (c) following a strict diet over time without transgressions; (d) social isolation and disruption of social functioning; (e) decreased quality of life; (f) feeling of superiority, control, and fulfillment; (g) feeling of guilt; (h) having a strong desire to be healthy; (i) being willing to spend more money for healthy food; and (j) having knowledge about healthy eating. When comparing these themes with the latest diagnostic criteria developed by Dunn and Bratman [71], some incongruences emerge. First, criterion B1 proposed by Dunn and Bratman (i.e., malnutrition, severe weight loss or other medical complications from the restricted diet) was not included in the areas inquired by the tools. In addition, four areas investigated by the tools are not included in Dunn and Bratman’s criteria, being (b) paying attention to calories and healthiness of food more than the pleasure of eating; (e) decreased quality of life; (i) being willing to spend more money for healthy food; and (j) having knowledge about healthy eating. The discrepancies between diagnostic criteria and diagnostic tools prove that a re-conceptualization of diagnostic criteria is needed and that the construction of a new tool needs to be based upon this re-conceptualization.

The studies that aimed to develop a diagnostic tool for ON acquired the information from the existing literature. Information on which to base the tools was obtained through literature reviews or analysis of case studies; thus, no new investigation of ON according to what people think of it today was performed. This means that what is known of ON comes from an initial definition of it, which was then repeatedly elaborated by several authors. However, being a social phenomenon, ON has evolved over time with the emergence of new trends and new communication channels. Therefore, a re-conceptualization of the phenomenon is necessary for a better understanding of the phenomenon.

Apart from a new interpretation of the phenomenon, another missing thing was the use of qualitative data collection techniques during the process of tool construction. The only qualitative technique adopted was the possibility to write comments on the questions analyzed during the Delphi method, adopted for developing the BOS [70]. Despite experts could write down their opinions, this was not enough for them to express their ideas and experiences. Therefore, interviews or focus group discussions should have been included to acquire in-depth information on how to diagnose ON. This was also suggested by Dunn et al. who underlined the importance of qualitative data for a comprehensive overview of diagnostic criteria for ON [9]. One thing common to all studies that developed a tool to assess ON was the lack of reporting of phases that explicitly involved multiple stakeholders in the research process. Tools seemed to be constructed mainly by academics, without the active involvement of health workers (e.g., psychologists, psychiatrists, and dietitians), patients, or people coming from the general population. The study conducted by [70] was the only one that involved “experts”, among which also health professionals, during part of the process of tool construction. However, the involvement of practitioners working in the field was considered a limitation of the study [70]. The involvement of academic and non-academic stakeholders during the phases on the research process is the core characteristic of transdisciplinary research (TDR) [72]. TDR is a useful methodology that allows taking into account multiple different perspectives, and thus re-conceptualizing a phenomenon going back to its roots. TDR is also crucial, because it allows to engage potential key users in the research process from the start, who can try to envision how results may be used [73]. In fact, one important step that needs to be taken when doing TDR is capacity building [74]. This means that key stakeholders need to be mobilized in order for them to be able to identify and to address their own needs [74]. Involving health professionals is, therefore, crucial, as they will use the tool in future to assess the presence of ON. This would allow them to be critical and to help constructing a tool that can be effective in diagnosing ON.

Three strengths of this review can be pointed out. First, it adopted a systematic search on three databases, which provided a wide range of articles. Second, a critical analysis was performed not only considering the studies that developed a tool for diagnosing ON, but also the studies that used a tool to diagnose ON. Finally, it critically analyzed the methodologies employed to develop the tools, thus providing useful information for a future construction of a new diagnostic tool. It is also necessary to indicate three limitations: the exclusion of some articles that did not meet language requirements, the impossibility of comparing all the characteristics of all the tools, due to the fact that some had been widely used over the years, while others were relatively new, and the non-inclusion of the papers published in 2019 (after the search) such as adaptation of the DOS to Chinese [75]. Overall, this review may be a starting point for developing a new tool for ON that can overcome the methodological weaknesses of previous studies. Therefore, suggestions for a future tool construction are: (a) starting from a modern re-conceptualization of ON, according to multiple perspectives; (b) adopting qualitative data collection techniques to gain insights into how to diagnose ON; and (c) actively involving multiple and diverse stakeholders during the phases of the study process.

## Conclusion

Orthorexia nervosa is a disordered eating pattern, whose prevalence rates are often contradictory, due to the use of different diagnostic tools. This literature review analyzed the studies that constructed diagnostic tools for ON, and the studies that used these tools to assess the presence of this disordered eating pattern. What emerged is that there are disagreements on the conceptualization of ON, which influence they way diagnostic tools are constructed. Some methodological weaknesses of the study that constructed the tools have also been pointed out, which may be taken into consideration when developing a new diagnostic tool for ON.
